# Evaluation and Improvement of Employee Performance with respect to Health, Safety, and Environment (HSE) Factors: A Case of Complex Transport Construction Project

**DOI:** 10.1155/2023/1741886

**Published:** 2023-08-26

**Authors:** Xinying Zhang, Yuanqing Wang, Yanan Gao, Yuanyuan Liu, Shuo Feng

**Affiliations:** ^1^College of Transportation Engineering, Department of Traffic Engineering, Chang'an University, Xi'an 710064, China; ^2^Guangdong University of Technology, Guangzhou 510006, Guangdong, China; ^3^Hebei Provincial Communications Planning and Design Institute, Shijiazhuang 050021, Hebei, China

## Abstract

Risk control in complex transport construction is complicated due to the dangerous nature of high variation and unpredictability. Most of the current research analysis focuses on the health, safety, and environment (HSE) risk assessment and employee performance evaluation, which neglects the impact of HSE risks on employee performance. Consequently, this research develops a framework to evaluate employee performance and identify key factors affecting performance. The employee performance indicators and HSE indicators are established by reviewing related literature. Using data from questionnaires, an artificial neural network- (ANN-) based model of employee activity effectiveness is then developed to evaluate employee performance. Sensitivity analysis is implemented to determine the key factors affecting employee performance. The Hong Kong-Zhuhai-Macau Bridge, a large-scale cross-sea channel project, is taken as a case study for validation. The model results show that the employees are satisfied with the effect of HSE management in general, but the psychological stress they perceive becomes large. The indicators of risk control and employee participation positively impact employee performance, while job satisfaction has a negative impact on performance. These findings indicate that operators should pay more attention to employees' psychological perception of work and form a standardized process management and control plan to prevent cumbersome processes from increasing employees' workload. This study helps construction systems and managers to identify the areas of strengths and weaknesses in their HSE management. The research only focuses on the impact of HSE risks on managers' performance in the complex transport construction project. In the future, further engineering projects and employee performance in composite scenarios can be investigated to improve the overall productivity.

## 1. Introduction

The transport industry has proven to be an indispensable component in guaranteeing the smooth running of socioeconomic activities by providing public services for social production and people's lives. Complex transportation construction projects (such as the Hong Kong-Zhuhai-Macau Bridge) face great challenges in risk control and safety management due to the dynamic complexity [1], sudden and variable nature of risks, and uncertain weather conditions [2]. Compared to other infrastructure construction (e.g., industrial sector), the workplace and production environment of transport construction is not fixed, and personnel and facilities possess mobility. Transport engineering construction is typically exposed to physical hazards (such as long-term exposure to high-temperature environments, noise, and vibration), chemical hazards (dust and fumes from emissions), and ergonomic hazards (such as prolonged sitting and repetitive motion of hands and arms) [3]. In comparison to traditional transport projects (e.g., subway construction), the complex transport construction project (the Hong Kong-Zhuhai-Macau Bridge) is a rare mix of sea-crossing channels, bridges, tunnels, and islands. Although its construction process is similar to that of civil engineering, the high variation and unpredictability of ground and hydrogeology make the construction process face many uncertainties and unknown factors. In addition, the long construction period, high construction cost, and complicated cross-operations make it particularly risky [4]. All these characteristics bring great challenges to the risk management of complex transport projects.

Health, safety, and environment (HSE), as a systematic and integrative management system developed in the 1980s, aims to assure safe production, reduce risks, prevent accidents, and achieve sustainable development [5]. Integrating occupational health risks, safety risks, and environmental risks in production processes, HSE has been gradually applied in high-risk industries, such as petroleum enterprises [6], paint manufacturing facilities [7], gas refineries [8], petrochemical plant [9], and oil and gas transportation plant [10]. However, there is a shortage of research on whether HSE management systems can be applied to the unusual transport project with the cross-sea channel, bridge, tunnel, and island mixed.

In the field of HSE risk assessment, scholars have done considerable effort using many mature methods and new technologies. Risk assessment techniques used in the field of HSE generally calculate the risk value depending on the probability and severity factors [11]. In the literature, the analytic hierarchy process (AHP) method has been frequently used to evaluate the factors in terms of contribution to the risk and analyze the priorities of these factors [12, 13]. To realize more accurate identification, some theories are combined in the risk assessment process, such as the fuzzy AHP [14], nonlinear fuzzy AHP [15], Pythagorean fuzzy AHP, and fuzzy inference system [11]. Moreover, as a method supported by new technology, machine learning (ML) has obvious advantages in analyzing uncertain risk factors. To investigate the relative importance of risk factors, random forests (RF), stochastic gradient tree boosting (SGTB) [16], Naive Bayesian classifier [17], and support vector machine (SVM) [18] are applied to predict the risk and the possible impact. Additionally, artificial neural network (ANN) has also been used to evaluate the risk factors because they can automatically adjust their weights and capture complex nonlinear relationships [8, 19]. In general, research on HSE risk assessment has focused on predicting the probability of risk occurrence and the impact caused by the risk, using classical or intelligent algorithms.

Minimizing human-caused risks is crucial to project construction and operation management due to the hazardous nature of complex transportation construction. The performance evaluation is of great importance considering that it can help reduce human-caused risks [20]. Performance evaluation aims to understand the positive and negative aspects of their performance, so as to maximize the organization's productivity. Based on monitoring of performance, it can be determined how risk management policy and plan must be improved [21]. In previous works in the field of performance evaluation, researchers have employed different approaches to identify key performance indicators [8, 20, 22–24]. However, there is a lack of research to assess the impact of HSE risk factors on employee performance, which is one of the purposes of this paper. HSE risk factors contain many indicators defined by experts (e.g., 28 indicators are defined by China National Petroleum Corporation). It requires much additional resources to evaluate each HSE risk indicator without identifying the key variables affecting performance. The Pareto principle, commonly known as the 80/20 rule, states that just 20% of the elements have an important bearing on performance in comparison with the other 80% elements [6]. Consequently, it is essential to develop a rigorous methodology to identify the key risk indicators that have an impact on performance. On the basis of this, this study examines the applicability of an HSE management system on an unusual and complex transportation project. Based on an intelligent methodology, the impact of HSE risk factors on employee performance is evaluated. This study helps managers to grasp the risk factors affecting employee performance and contributes to the improvement of risk management in complex transport construction projects.

The rest of this paper is organized as follows. Some relevant research and methods will be reviewed in the next part. This is followed by methods of the employee HSE performance evaluation. Next, the results and discussions are presented in succession. Finally, the paper is summarized.

## 2. Literature Review

### 2.1. Complex Transport Projects

In recent years, complex transport projects have been constructed in massive numbers as an essential tool to support society travel. Complex transport projects involve multiple disciplines such as roads, bridges, railroads, and tunnels. A very high level of long-term safety risk exists in construction since it requires the collaboration of thousands of workers and sophisticated technologies. Its construction process is generally a substantial, expensive, and one-time construction project with complicated and varied construction sites. In addition to complex engineering systems, the construction usually concerns complex hydrogeological conditions, fluctuating working environments, considerable employee mobility, and lengthy construction cycles. All of these factors increase the risk of safety incidents and work-related injuries during all phases of construction [25, 26]. The Hong Kong-Zhuhai-Macau Bridge, as a typical representative of a complex transport project in China, contains three navigable bridges, an undersea tunnel, and four artificial islands. With a total length of 55 km (including 22.9 km of bridge and 6.7 km of undersea tunnel), the Hong Kong-Zhuhai-Macau Bridge is renowned for its enormous construction scale, exceptional execution difficulties, and top-notch technology. Its entire length is 55 km, which includes 22.9 km of bridge work and 6.7 km of undersea tunnel. Due to complicated meteorological and hydrological conditions, crossing natural ecological reserves, the various technical standards of Guangdong, Hong Kong, and Macau, frequent personnel movements, and intensive high-risk operations, the construction process is highly susceptible to safety and environmental accidents [1–3]. Therefore, the search for efficient risk control and personnel management methods is a top priority for the construction of complex transport projects.

### 2.2. HSE Regulations

Health, safety, and environment, usually referred to as HSE, was proposed initially by the International Organization for Standardization Technical Committee in 1976. It aims to assure safe production, decrease accidents, and achieve sustainable development [6]. Based on the continuous improvement cycle of Deming, the HSE management follows the concept of plan-do-check-action (PDCA) to boost system performance. By constantly learning from past experiences and effective benchmarking of competitors, the risk of human, environmental, and capital resources can be minimized significantly [27]. The International Organization for Standardization (ISO) has formulated a series of international standards (e.g., ISO-9000 and ISO14001) to strengthen the quality and environmental management in high-risk industries. Since then, HSE implementation has been prioritized in many countries. For example, the Voluntary Protection Program (VPP) in the United States, an Occupational Safety and Health Administration (OSHA) initiative, encourages companies to prevent workplace injuries and illnesses through hazard prevention and control, worksite analysis, training, and collaboration between management and workers. Also, Chinese government promulgated some HSE standards (e.g., GB/T24001-1996, GB/T 28001-2001, Q/SY1002.1-2007, and Q/SY1002.2-2008) to ensure the management efficiency [6]. In some European Union (EU) member states, HSE management programs have also been developed to improve their safety performance [28]. All these studies and applications have proved that HSE is frequently used in high-risk construction projects to eliminate or decrease safety injuries, adverse health influences, and hurt to the environment [8].

### 2.3. HSE Practices in the Construction Industry

HSE system has played an important role in risk and safety management in construction industry in past decades. Many researchers have studied the HSE implementation and evaluation to improve safety in construction. Saksvik and Nytro [29] presented an implementation of internal control (IC) of health, environment, and safety (HES) in Norwegian enterprises, involving systematic actions that reduce stress and occupational ill health to prevent injuries in workplace. Torp and Moen [30] presented the effects of implementing occupational health and safety (H&S) management system. The results indicate that positively changing the occupational H&S management may positively change workers' satisfaction, psychosocial work environment, and health-related behavior. Duijm et al. [28] demonstrated that HSE management would benefit greatly from guidance on how to utilize current management systems effectively and also from the further development of meaningful safety indicators. Moreover, researchers have considered incorporating further elements to demonstrate the impact on HSE management. Azadeh et al. [31] have cooperated the ergonomics to the workplace including machine, job, and environment. Zarrin and Azadeh [32] evaluated and analyzed the impacts of resilience principles on HSE management system by using Z-number cognitive map. The aim of their study was to gain insight into different aspects of HSE and how to improve safety by performing HSE.

### 2.4. The Impact of HSE Risk Factors on Employees' Performance

Nowadays, much attention has also been given to evaluating employee performance and identifying the HSE factors that affect performance because of the fact that more than 80% of incidents are attributable to illegal operations, low-security awareness, and poor technical skills of employees [33]. In other words, accidents caused by employee errors can be reduced by improving the management of the risk factors that affect employee performance. Shikdar and Das [34] have reported that a significant correlation exists among productivity indicators and health and organizational attributes. This inspires that certain HSE risks may significantly influence the overall employees' performance and therefore must be considered and designed with more attention. Yan et al. [6] applied the improved fuzzy comprehensive evaluation method to identify the key HSE factors. Six factors of leadership and commitment; health, safety, and environment mission; competence, training, and awareness; control of documents; HSE management of contractor and suppliers; and incident/accident report, investigation, and management were identified as key performance indicators. Considering the uncertain nature of incidents, data envelopment analysis (DEA) models were employed to identify the efficient units by Pashapour et al. [9]. Results show that preparedness and planning is one of the most important factors in petrochemical plant. Using the fuzzy cognitive map (FCM) approach, Asadzadeh et al. [35] concluded that instructions and education, familiarity with organization's rules, and proper communications most contribute to improve workers' safety, satisfaction, and productivity. Besides, researchers have frequently used artificial neural networks (ANNs) for performance evaluation in recent years because they are superior to conventional methods in determining complicated relationships and can automatically adjust their weights [36–38]. Azadeh et al. [39] and Azadeh et al. [24] proved that ANN provides an excellent solution regarding the assessment of performance.

Understanding and structuring employee performance factors are very essential tasks for organizations to encourage productive behaviors of employees as well as discourage those that are unproductive. Three factors are used frequently to reflect the employee performance, whether positive or negative. These factors are job satisfaction [20, 40, 41], HSE awareness [32, 42–45], and work stress [11, 20]. These factors have been a reliable representation of performance. The definitions and importance of these factors in the fields of HSE and performance management are briefly described below.

#### 2.4.1. Job Satisfaction

Job satisfaction has been defined in many forms. The traditional definition of job satisfaction focuses on the feelings that individuals have about their job experience [8]. Job satisfaction is the level of contentment employees feel about their work, which can affect performance [46].

#### 2.4.2. HSE Awareness

HSE awareness refers to personnel that have a duty to be aware of both their own present status and the status of the defenses in their company [20]. HSE awareness is considered to be an important indicator for predicting and assessing the performance that may affect the proper functioning of the system [20, 47]. Also, HSE awareness is significant for the evaluation of the trade-offs between safety and production in HSE management.

#### 2.4.3. Work Stress

Work stress is an adverse physical and emotional response that occurs when the workload is excessive [31, 48]. Work stress is both a powerful motivator and a negative factor that affects employee performance and occupational health. Managing this factor is the responsibility of both employees and employers. Effective work stress management can help improve employee productivity [20].

## 3. Methods

**Figure 1:**
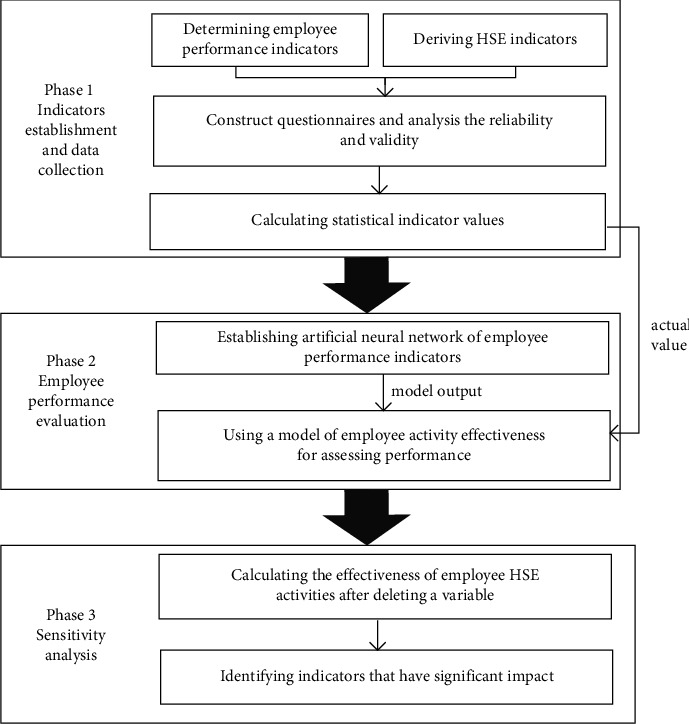
Flowchart of the methodology framework.

In this study, we use an intelligent methodology to complete the evaluation of employee performance. The whole methodology consists of three parts: (1) to establish employee performance indicators and HSE indicators; (2) to construct an ANN model to explore the relationship between employee performance indicators and HSE indicators, whose output is used as part of the employee activity effectiveness; and (3) to perform a sensitivity analysis to determine the key factors affecting performance. The flowchart of the proposed methodology is presented in Figure 1.

### 3.1. Indicator Construction and Data Gathering

This paper utilizes an intelligent methodology for employee performance evaluation in a complex transport construction project. Based on the literature review, three indicators reflecting employee performance are established (i.e., job satisfaction, HSE awareness, and work stress). The HSE indicators are selected to observe employees' responses. A total of fifteen items are derived, which include important elements in international standard practices and evaluation models developed by Zohar [42], McDonald et al. [49], Høivik et al. [50], Yan et al. [6], and Pashapour et al. [9].  Table 1 lists the references from which the indicators are derived.

In designing the questionnaire, we tried to cover fifteen HSE indicators and three employee performance indicators derived from the references. The preliminary questionnaire is distributed to a six-member group of experts. The group of experts consists of four HSE experts and two managers from HSE administrative, safety and environmental department, and other research institutions. All of the experts consulted have more than ten years of experience in safety engineering, civil engineering, or structural engineering.  Table 2 shows the brief description of six experts in the questionnaire presurvey. It should be noted that, based on the comments returned to managers in the presurvey, some questions are added, modified, replaced, or deleted, and the final version of the survey questionnaire is shown in Table 3.

As a complex transportation construction project, the Hong Kong-Zhuhai-Macau Bridge covers several types of work, including bridges, island tunnels, and transportation engineering. As the project involves numerous aspects (including survey, design, consulting, construction, and supervision), with various types of risks, most of the grassroots personnel are responsible for the construction of the project and lack a thorough understanding of the HSE management system. In order to ensure the reliability and accuracy of the information obtained, we set the respondents as HSE managers of different departments, such as the Safety and Environmental Protection or Operation and Management Department, because the managers are relatively fixed and they were more knowledgeable about the HSE management implementation process. The respondents were trained during the survey process to accurately capture the content of the questionnaire and improve the quality of questionnaire filling. There were a total of 149 HSE management personnel working on the project, and 120 of them filled out the questionnaires based on the respondents' willingness. The margin of error determined according to the following statistical method is 0.089 (equation (1)), so the dataset size is considered reasonable.

(1) $$\begin{array}{c} n=\frac{{\left[Z_{1-\left(1/2\right)\alpha }\right]}^{2}\sigma^{2}}{r^{2}}\text{,} \end{array}$$

where *r* is the margin of error or precision; *σ* is the standard deviation assumed to be 0.5; and *Z*_1−(1/2)*α*_ is the standard normal statistic corresponding to the (1 − *α*) level.

The questionnaires have been distributed in papers, and the required data are collected face to face. The degree of each answer takes the form of a percentage system from 0 to 100 points (0 = quite disagree; 100 = quite agree). The final 96 questionnaires are adopted after removing the ineffective questionnaires. Despite the small dataset, the ANN-based model can still be applied because there are no strict criteria on the dataset size of ANNs. For example, in the previous studies of engineering management that used questionnaires as a data source for ANN, the dataset sizes were 250 in Patel and Jha [19], 115 in Azadeh et al. [31], 85 in [58], 70 in Azadeh et al. [8], and 40 in [59], respectively.

The demographic profile of the effective respondents is presented in Table 4. The dimensionless normalization is performed on the data collected, and all the data are transformed into the interval [0, 1], and the difference in the magnitude of the data in each dimension is eliminated. In order to evaluate reliability, Cronbach's alpha of the questionnaire is measured, as shown in the following formula:

(2) $$\begin{array}{c} {\text{Cronbach}}^{\prime }s\;alpha=\frac{n}{n-1}\left(1-\frac{\sum s_{i}^{2}}{S_{t}^{2}}\right)\text{,} \end{array}$$

where *n* represents the number of problems; *s*_*i*_ represents the standard deviation of the problem; and *S*_*t*_ represents the total standard deviation. Cronbach's alpha ranges from 0 to 1. Generally, alpha greater than 0.8 reflects a high internal consistency of the questionnaire; alpha in the range of 0.6–0.8 reflects a better internal consistency; a smaller value of alpha than 0.6 indicates that the internal consistency is poor [60].

This paper analyzes the validity of the questionnaire through a content validity ratio (CVR). Experts are invited to evaluate the question to the evaluation of the indicator as “not essential,” “useful but not necessary,” or “essential.” If more than 50% of the experts believe that the problem is essential, the item has certain content validity. CVR can be calculated using the following equation [61]:

(3) $$\begin{array}{c} CVR=\frac{n_{e}-N/2}{N/2}\text{,} \end{array}$$

where *n*_*e*_ is the number of experts who choose “essential” and *N* is the total number of experts.

### 3.2. Employee Performance Evaluation

Research has shown that performance can be calculated by judging the effectiveness of activities, such as accomplishing goals and adopting strategies. The US National Audit Office defines performance as the effectiveness of strategies used to solve or mitigate targeted problems. Brown and Pyers [62] believe that performance evaluation is an activity that corrects the behavior of the subject by judging the effectiveness of activity. The HSE performance was evaluated through the calculation of the employee activity effectiveness in this paper. Effectiveness is related to the difference existing between the actual value (i.e., target) and the maximum value [20, 31]. In other words, effectiveness is determined by comparing what a process or employee can produce with what they actually produce (the maximum and actual produce). The maximum value refers to employees' behavior that exceeds the organization's requirements and job description [63]. The actual value can be obtained from the questionnaire data [20].

#### 3.2.1. Construction of an Artificial Neural Network

An ANN is applied to explore the relationship between fifteen HSE indicators and employee performance indicators. The following steps are considered for the development of the ANN-based model:

1. Determine the input and output variables: As stated previously, the three indicators of employee performance (job satisfaction, work stress, and HSE awareness) take turns as the output variable; the other two indicators of employee performance and fifteen HSE indicators are used as input variables in the development of the model [8, 64].
2. Select architecture: The neural network chosen is a multilayered feed-forward network with backpropagation (BP) because it is efficient for performing any linear or multivariate arbitrary nonlinear computation. The training is carried out using the MATLAB Neural Network (NN) toolbox. One hidden layer may be enough, especially when it is not overladen with too many hidden nodes [65]. So, one hidden layer is chosen and the number of neurons in this layer is decided in the learning process by trial and error.
3. Training and testing of data: The number of test sets and training sets is determined empirically. Out of a total of 96, 86 (90%) and 10 (10%) datasets are used for training and testing of the network in this paper, respectively. In the previous studies, Azadeh et al. [31] used data from 100 (87% of 115) to train the network and 15 (13% of 115) to test it. In Jha and Chockalingam [58], 65 user's datasets were used for training and the remaining 20 datasets were used for validation. Maya et al. [59] used data from 32 (80% of 40) projects to train the network and 8 (20% of 40) to test the network.
4. Determine the number of neurons in a hidden layer: A fewer number of hidden-layer neurons result in underfitting problems, while a greater number of neurons lead to overfitting problems. According to a rule of thumb, the number of neurons in the hidden layer should be less than twice the number of neurons in the input layer [66]. Therefore, several trials are carried out with different numbers of neurons in the hidden layer, starting from 1 neuron and progressively increasing up to 50 neurons. The optimal number of neurons in the hidden layer is decided based on the least mean absolute percentage error (MAPE).
5. Select training parameters: Three transfer functions universally applied in ANN are log-sigmoid (logsig) function, tan-sigmoid (tansig) function, and linear transfer function (purelin). The combination of logsig-purelin function and tansig-purelin function is chosen as the transfer function of the model considering the nonlinear question involved in this paper. Seventeen commonly used training functions are selected to train the neural network in this paper (Table 5). The expected error (goal) of the network training result is set to 0.05; the learning step (lr) is set to 0.01, and the maximum training number (epochs) is set to 5000.
6. Finalization of the ANN model: A total of 102 neural networks (17 (training function) *∗* 2 (transfer function) *∗* 3 (employee performance indicators)) are constructed to acquire the optimal neural network based on the minimum principle of MAPE. As shown in Table 5, the optimal ANN model for job satisfaction has 14 hidden layer neurons, with the MAPE at 0.0439. The optimal ANN model for working stress has 13 hidden layer neurons, with the MAPE at 0.0572. The optimal ANN model for HSE awareness has 23 hidden layer neurons, with the MAPE at 0.0713.

#### 3.2.2. Calculation and Analysis of the Effectiveness

After finding the optimal neural network, the model output is computed and the employee activity effectiveness is measured according to the following process.

The datasets were put into the optimal neural network model for job satisfaction, and the errors between the actual value (*P*_actual(*i*)_) and the model output (*P*_model(*i*)_) for all dataset were calculated as follows [20, 24]:

(4) $$\begin{array}{c} E_{i}=\left|P_{actual\left(i\right)}-P_{model\left(i\right)}\right|,i=1,2,\ldots ,n\text{.} \end{array}$$

Assume that *k* has the largest error *E*_*i*_ and therefore:

(5) $$\begin{array}{c} E_{k}=\max_{i=1,2,\ldots ,n}\left(E_{i}\right)\text{.} \end{array}$$

The value of *E*_*k*_′ which is the most remarkable difference between the maximum job satisfaction and general job satisfaction is defined as *E*_*k*_:

(6) $$\begin{array}{c} {E_{k}}^{\prime }=E_{k}\text{.} \end{array}$$

Therefore, the difference *D*_*i*_ between the general job satisfaction and the maximum job satisfaction of each kind of data can be calculated by the following formula:

(7) $$\begin{array}{c} D_{i}=\frac{{E_{k}}^{\prime }*P_{model\left(i\right)}}{P_{model\left(k\right)}},i=1,2,\ldots \text{.} \end{array}$$

The job satisfaction effectiveness *P*_*i*_ of data *i* is the ratio of the actual job satisfaction of the employees to the maximum job satisfaction of employees. The actual job satisfaction is *P*_actual(*i*)_, and the maximum employee satisfaction can be determined by *D*_*i*_ and *P*_model(*i*)_:

(8) $$\begin{array}{c} P_{i}=\frac{P_{actual\left(i\right)}}{D_{i}+P_{model\left(i\right)}},i=1,2,\ldots ,n\text{.} \end{array}$$

Similarly, the work stress effectiveness *W*_*i*_ and the HSE awareness effectiveness *H*_*i*_ of employees can be calculated based on the optimal neural network model. Since it is difficult to determine the importance of the three indicators, we only need to simply weigh the effectiveness of these three indicators and finally obtain the effectiveness of the employees' HSE activity *A*_*i*_:

(9) $$\begin{array}{c} A_{i}=\sum \left(\frac{1}{3}P_{i}+\frac{1}{3}W_{i}+\frac{1}{3}H_{i}\right),i=1,2,\ldots ,n\text{.} \end{array}$$

According to the research results of Amir-Heidari et al. [21], the value of employee HSE activity is divided into four levels: [0, 0.6) is unqualified, [0.6, 0.8) is qualified, [0.8, 0.9) is good, and [0.9, 1] is excellent. In other words, when the employee HSE activity effectiveness is between [0, 0.6), the performance level is unqualified; when the employee HSE activity effectiveness is between [0.6, 0.8), the performance is qualified; the performance is good when HSE activity effectiveness between [0.8, 0.9); the performance is excellent when employee HSE activity effectiveness is between [0.9, 1].

### 3.3. Sensitivity Analysis

Different factors have different contributions to employees' performance. Identifying indicators that have a significant impact is beneficial to improve management performance, which can be achieved by sensitivity analysis. Sensitivity analysis identifies the degree to which each of the input variables contributes to each of the output variables [19, 38]. To determine the key factors affecting the employee HSE activity effectiveness, the 17 input variables in the model are deleted successively; the optimal model is applied to recalculate the effectiveness. In this paper, the confidence level *α* is set to be 0.05, and the degree of freedom is 95. To determine whether a variable has a significant impact on the effectiveness of employees' HSE activities (*H*_0_: *μ*_*F*_ = *μ*_*i*_, *H*_1_: *μ*_*F*_ ≠ *μ*_*i*_), we use SPSS to compare the effectiveness of the entire variable (*μ*_*F*_) and the effectiveness after deleting the variable (*μ*_*i*_) to perform a paired-sample bilateral *t*-test [20, 22]. The *P* value gives the probability of observing the test results under the null hypothesis. If the *P* value is less than 0.05, the null hypothesis is accepted, which means that the deleted variables' effect on the employee HSE activity effectiveness is not significant. On the contrary, it means that the deleted variable has a significant impact on the effectiveness. If the effect is significant, then the paired sample unilateral *t*-test is employed to determine whether the effect is positive or negative (*H*_0_: *μ*_*F*_ > *μ*_*i*_, *H*_1_: *μ*_*F*_ < *μ*_*i*_). If the null hypothesis is accepted, it means that the corresponding indicator has a better effect, that is, the factor has a positive impact on the effectiveness because its deletion reduces the effectiveness; if the null hypothesis is rejected, it means that the factor harms the effectiveness of employee HSE activities.

## 4. Results

After descriptive statistical analysis of the questionnaire, the effectiveness of employee activities is calculated through the constructed model. Then, sensitivity analysis is used to determine indicators that have a significant impact on effectiveness, which allows managers to determine the key factors affecting employee performance in complex transport construction projects.

### 4.1. Statistical Analysis of the Questionnaire

The final questionnaire designed was distributed to 120 managers of the project and the contractors in different positions, and 96 available copies obtained finally with poor-quality questionnaires were excluded. After standardizing and unifying the questionnaire's data, Cronbach's alpha and CVR are calculated to verify the questionnaire's reliability, as shown in Table 6. Cronbach's alpha for all indicators is greater than 0.7, and CVR values are greater than 0, indicating that the final questionnaire has an acceptable level of reliability in complex transport construction projects. Then, for each kind of data, the average value of the problem corresponding to each indicator is counted to analyze. A descriptive statistical analysis of each indicator is performed by SPSS, sorted by mean in descending order, as shown in Table 6.

Table 6 shows that the average value of 15 HSE management indicators is basically above 0.9 (accident investigation report and handling excluded), reflecting the excellent performance of the HSE management process in the project. The average value of the accident investigation report and handling index is only 0.8, which is significantly lower than that of other management indicators. The reason may be that the complex transport construction project involves much technology and personnel, and the accident analysis and the determination of responsibility are more detailed than previous management. Besides, the means of employee performance indicators (work stress, HSE awareness, and job satisfaction) are generally lower than those of the HSE management indicators, demonstrating that employees are not satisfied with the HSE management. In particular, HSE awareness and job satisfaction indicators are far lower than other indicators, reflecting that employees are not aware of HSE management's importance and may not be adaptable after applying HSE management in complex transport construction projects.

### 4.2. Employee Performance Evaluation

**Figure 2:**
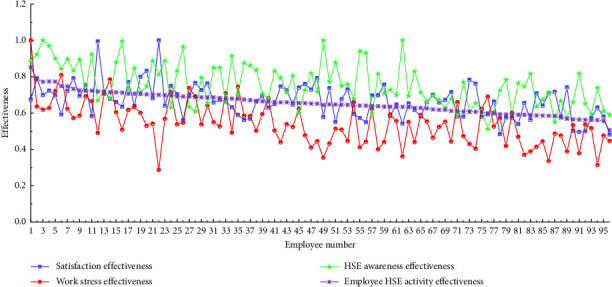
Employee HSE activity effectiveness.

As discussed in the previous section, employee performance is divided into three components: job satisfaction, HSE awareness, and work stress, and employee performance is quantified through employee activity effectiveness. We calculate the three performance indicators' effectiveness and the total employee activity effectiveness according to formulas equations (4)–(9) as shown in Figure 2. The effectiveness of job satisfaction and HSE awareness is mostly concentrated above 0.6, indicating that employees' actual job satisfaction and HSE awareness are effective. In other words, in the implementation of the HSE management system of the project, most respondents are satisfied with the psychological perception of work and have sufficient awareness to work in the HSE management mode. This suggests that the respondents' sense of work stress is rather high throughout the HSE management system's implementation. This perception may be a result of the increased effort brought on by more stringent safety management.

Although the three employee performance indicators show different levels of effectiveness, the employee activity effectiveness of 80.2% of employees is not less than 0.6; that is, the performance of most employees is above the qualified level, indicating that employees psychologically identify with the implementation of the HSE management system in the construction of complex transport construction projects.

To verify the relationship between job satisfaction effectiveness, work stress effectiveness, and HSE awareness effectiveness, the Pearson correlation coefficient is used, as shown in Table 7. It indicates that the correlation between the effectiveness of the three employee performance indicators is weak, and their interaction is negligible, which demonstrates that the three performance indicators can objectively reflect the level of employee HSE activity effectiveness.

**Figure 3:**
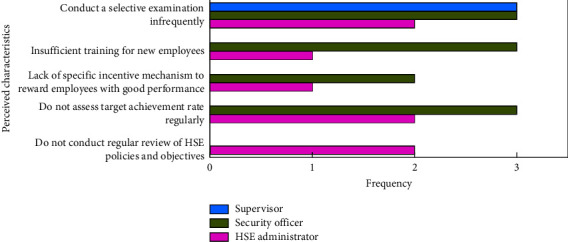
Perceived characteristics of unqualified employee activity effectiveness.

To understand the employees' reasons for unqualified activity effectiveness, their perception of the HSE management is reported statistically (Figure 3). The results show that low-performing employees think that there are many disadvantages, such as few checks, insufficient training and education for new entrants, lack of incentive mechanisms, and no regular evaluation and reviews of the objectives. Likewise, Koehn et al. [67] suggested that continuous self-inspection at the site, education for employees, and use of a safety analysis are vital to maintaining a safe project. Azadeh and Zarrin [20] stated motivational works enable to increase their human resource productivity. Therefore, employees with unqualified activity effectiveness may be caused by the focus on these factors in their job responsibilities. The HSE administrators' perception of failure to conduct regular assessment and review of the goals may be the reason for the low activity effectiveness. Similarly, the low effectiveness of security officers may be due to their commitment to accident prevention and their responsibility for regular training and education. Besides, the supervisors' attitude to the low rate of spot checks implies that the project may not be strict with on-site supervision and inspection.

**Figure 4:**
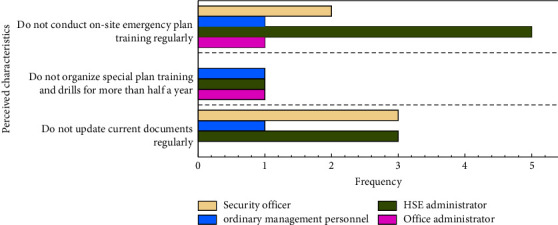
Perceived characteristics of unqualified employee work stress effectiveness.

In particular, almost 66.7% of employees' work stress effectiveness is lower than 0.6, implying that employees are under greater pressure when implementing the HSE management system. To find out the cause of greater pressure, the employee perception characteristics with the effect of unqualified work stress were proposed (Figure 4). The results indicate that employees with heavy pressure mainly think that the project has not regularly updated and revised HSE laws and regulations, and the frequency of organizing emergency plan training and drills was low. Especially, employees from multiple positions of HSE administrators, security officers, office administrators, and ordinary management personnel all believe that the emergency plan training is infrequent, probably because employees take accident prevention and emergency exercises seriously in the large multitask construction.

### 4.3. Sensitivity Analysis

To identify the key indicators affecting employee performance, sensitivity analysis is employed by recalculating the effectiveness after deleting input variables successively. The paired sample *t*-test results are shown in Table 8.

The bilateral *t*-test results indicate that the *p* values of the risk control, employee participation, and job satisfaction are 0.375, 0.514, and 0.057, respectively, which are greater than the significance level of 0.05 so that they have a considerable impact on employee activity effectiveness. HSE organization, responsibilities, and authority have a little impact on effectiveness. The remaining variables have little impact on effectiveness. Therefore, when applying the HSE management system to complex transport construction projects, full attention should be paid to risk control, employee participation, and employee job satisfaction.

Besides, the unilateral *t*-test results show that the risk control system and employee participation have a positive impact on the effectiveness of employee activities, as the effectiveness is reduced after removing the risk control or employee participation variables (*μ*_*F*_ > *μ*_*i*_). This implies that the HSE management system's introduction made the project more stringent for risk control and increased employees' sense of participation. However, job satisfaction has a negative impact on the effectiveness because the removal of job satisfaction variables increases the effectiveness (*μ*_*F*_ < *μ*_*i*_). This variation reflects the lack of sufficient attention to job satisfaction in the project [20]. Job satisfaction, as an important factor in improving effectiveness, usually reflects the individual's response to the job experience and the contentment employees feel about their work [46]. As a result, it can be inferred that the project did not pay enough attention to the employees' experience when implementing the HSE management system. This result gives us insights into managing employees. Knowing the physical and mental workload and stress imposed on employees can lead to more accurate planning in order to improve job satisfaction. By focusing on motivational works and reasonable workload, the employees enable to increase their productivity, which ultimately results in a notable improvement in work performance.

## 5. Discussion

### 5.1. Employee Performance Evaluation

The findings of the performance evaluation revealed that 80.2% of the employees performed at a qualified level, showing that the majority of them thought the application of HSE management systems in complicated transport construction projects was effective. According to the responses provided by these employees in the survey, the implementation of HSE management has increased the efficiency of project risk control and safety management. A precise control of the entire process is achieved in HSE management through the risk source identification, risk evaluation, and control. It was discovered that 19.8% of employees had unqualified performance, frequently as a result of a perceived deficiency in ongoing self-auditing and adequate training and education. In fact, in the dynamic work environment of the construction industry, the attitude of construction personnel towards safety directly influences their behavior on site. There is a need for regular training and occasional spot checks on safety practices and risk prevention to reduce the occurrence of personnel-related accidents. Additionally, a large cause of unqualified employee performance is the increased work pressure, as reflected by the fact that nearly 66.7% of employees have a work stress effectiveness below 0.6. These stressed employees may consider the workload or work difficulty during the construction of the project to be high. Considering the project background, the construction of the project faces complex conditions such as typhoons, rainstorms, thunder and lightning, high temperatures, and sea streams, causing operational difficulties, and danger factors are higher than those of traditional transport construction projects. These could be the causes of the increased work pressure.

### 5.2. Key Factors Affecting Performance

Sensitivity analysis identified three factors (risk control, employee participation, and job satisfaction) as key indicators that affect employee performance. As one of the most important factors of project management, risk control is regarded as an influential factor affecting system performance in industries such as gas refineries and power plants [27, 35]. The main factors that affect the risks of engineering construction include personnel safety knowledge, effective risk management systems, risk identification and monitoring, and appropriate specifications and standards [68]. The risk control has a positive impact on employee activities' effectiveness in this project, which means that the risks in the project are well controlled generally during the implementation of the HSE management system. The project has significantly reduced the accident rate under the complex meteorological conditions and strict marine ecological environment protection requirements. This indicates that the subtle risk source identification, timely risk evaluation, and dynamic update of risk control management in the project construction process have effectively achieved risk prevention and control.

Employee participation, which is another significant factor according to the sensitivity analysis, has a positive impact on effectiveness in the case study. This is similar to the study by Alazzaz and Whyte [69], which stated a positive relationship between employee empowerment factors and productivity in off-site construction. It can be inferred that employees can recognize the importance of HSE management and actively participate in the HSE management process in this project. This may be because the project promotes full participation, encourages active management, and implements the HSE management concept at various stages. The multiple construction teams from bridges, subsea tunnels, and artificial islands are staggered in the limited sea environment, bringing significant personnel management challenges. In this project's HSE management process, the HSE concept is layered conveyed to each worksite and personnel through the method of publicity, training, inspection, and assessment, making employees grasp the HSE policy and objectives. In addition, commanders, professional technicians, and management personnel should participate in risk management and potential accident assessment and optimize HSE process management through uninterrupted practice and continuous improvement strategies of employees.

Interestingly, job satisfaction has a negative impact on performance in this project, which is inconsistent with the results of existing studies where job satisfaction has a positive impact on system performance and has been implemented appropriately in the petrochemical industry [70]. Since job satisfaction is the result of a combination of factors such as workload, attitude, and mental and physical conditions, this abnormal result reflects that the project lacks attention to the psychological state of employees during the implementation of the HSE system [71]. The cross-sea passage project strictly controls and inspects employees' operating procedures under the background of high construction risk and attaches importance to the occupational health examination of employees. However, the long-term monotonous offshore construction environment and severe weather conditions can easily cause psychological imbalances among employees. The project lacks direct and effective measures to alleviate psychological dissatisfaction during the construction process. In addition, during the construction of the project, severe education and economic punishment measures have been taken to control the unsafe behaviors of employees who violate the regulations, but the lack of incentives for outstanding employees may affect their psychology [71].

### 5.3. Development of the Framework for Evaluating Performance

This paper develops an intelligent framework to assess employee performance on HSE management systems and to identify key influencing factors in complex transport projects. As shown in Figure 1, the framework is composed of three stages. The first stage is to identify HSE indicators and performance indicators. The first stage of the framework is expected to determine the performance indicators and HSE indicators and gain responses about these indicators. In this stage, qualitative and quantitative knowledge about the details of HSE implementation in the organization is gathered, and performance-related employee perception is obtained. Although the selection of HSE indicators is mostly benchmarked against HSE regulations, the performance measurement of different construction fields may need its special considerations [21]. Therefore, the establishment of indicators needs to consider the characteristics and differences of construction projects. For instance, the hazards associated with transport construction projects are sudden and variable; therefore, HSE indicators and surveys should regularly identify the primary risk sources and shorten the time needed to control them. Additionally, there are other indicators (such as work security) that are taken into account in accordance with complex systems and dangerous circumstances, in addition to those mentioned in this research [20]. Of course, it is necessary to assess the correlation between these evaluation criteria in advance. The second stage is to construct an ANN-based evaluation model. The ANN model was chosen as the tool due to its superiority over other similar models such as regression models. Performance indicators can have both positive and negative effects on performance, for instance, job satisfaction is positive and work stress is negative. It is significant to emphasize that further study is needed on how to more effectively weigh performance indicators and integrate both positive and negative effects on performance. The identification of the crucial performance indicators is the third stage. In fact, most of the identified key factors are mainly based on expert knowledge and experience, or according to the score ranking of all indicators which is too cursory and arbitrary. The key factors identified in this paper can achieve the maximum efficiency improvement with the least cost. It should be noted that these identified key performance indicators may change with the increase of statistical data. This makes HSE performance evaluation more adaptable to changing circumstances.

### 5.4. The Implications of Findings

The results obtained in this study have several implications for policymakers. Construction managers should be aware that the most crucial elements in ensuring safe construction are those that relate to employees' operational and safety knowledge. HSE management systems' more rigid management guidelines and regulated operational procedures affect employees' traditional work practices while also adding to their workload. Therefore, only a relatively good safety management process is not enough, and the management of transport construction projects should also pay attention to the psychological state of employees, which can affect the operational effectiveness. Several actions should be made in this regard. To reduce the risk of overwork, employees' physical and mental stress should be periodically assessed. Additional means, such as psychological help and provision of personnel benefits and financial incentives, can also be used to increase employee motivation. Another important aspect is to strengthen employees' psychological acceptance of HSE management. As the management process implementers, employees must be made aware of the value and significance of the HSE management process. Moreover, it is important to understand the difficulty and cumbersome degree of the implementation process for employees and allow them to make suggestions on the system process. This facilitates the execution of the HSE management system and lessens the employees' unfavorable psychological reactions. Construction projects should introduce information technology equipment and facilities to assist in process management, reduce redundant workload, and improve management efficiency.

The results of this study help organizations to improve employee performance by managing key elements. To better implement HSE management in transport construction projects, organizations have a basic understanding of the impact of different factors through this paper. The findings of this study evaluation assist managers in developing a thorough grasp of the elements that influence worker performance and in enhancing their focus on the psychological well-being of workers. The proposed methodology can be applied to other similar construction projects to identify the most important and critical HSE elements of the construction process.

## 6. Conclusions and Recommendations

In this study, an integrated method is proposed to study the impact of HSE risk indicators on employee performance in a complex transport construction project, which is a supplement to the risk management knowledge system. Based on the questionnaire survey of HSE management professionals, an employee activity effectiveness model with an ANN is established to evaluate employee performance. Then, sensitivity analysis is applied to identify critical factors affecting performance for further reference. The results indicate that most employees' performance reaches a qualified level when implementing the HSE management system in the complex transportation construction project. The performance of 19.8% of employees is unqualified because they perceive that the project is not adequate for regular assessment of the objectives and new employee training, and the on-site inspection is infrequent. Sensitivity analysis finds that the indicators of risk control and employee participation positively impact employee performance, indicating that risk control is paid enough attention during the construction process, and the employees can actively participate in HSE management. However, job satisfaction has a negative impact on performance, which means that employees' perceptions of work are not being paid enough attention.

This evaluation's results provide managers and stakeholders with elements that HSE management needs to focus on in complex transport construction projects. Aspects like work pressure, incentive mechanism, and the usage of information technology are crucial for researchers and managers in terms of enhancing employee performance and effectiveness. Understanding the physical and mental loads and stresses of employees can help develop more accurate strategies to enhance health and safety conditions for employees [70]. Managers can encourage employees to proactively provide feedback on the physical quality to demonstrate the organization's support. In addition, soft measures such as appropriate reward and punishment mechanisms are highly applicable and may find a compromise between controlling costs and guaranteeing safety. For instance, safety incentives, which can be monetary or nonmonetary awards, are one of the proactive strategies used by managers to motivate employees to work safely [48]. Moreover, the application of diverse information and intelligent technologies may effectively address the safety issues in construction management. Information-based equipment and facilities should be introduced in transport construction projects to improve the quality and efficiency of risk control and safety management. Managers can further optimize information turnover and responsiveness through systematic planning, organization, and control of information resources.

However, this study still has some limitations. On the one hand, the number of questionnaires collected is relatively fewer because the subject of this article focuses on the HSE full-time manager of the Hong Kong-Zhuhai-Macau Bridge. In future research, grassroots employees can be used as survey objects to study the implementation of the HSE management system. On the other hand, it is important to further explore the obstacles while using the HSE management system for a construction project with a single scene.

## Figures and Tables

**Table 1:** Sources of fifteen indicators.

Indicators	Sources
Management system and procedures	Geller [43]; Isla Díaz and Díaz Cabrera [40]; Guldenmund [51]; Flin et al. [41]; Guldenmund [52]; Høivik et al. [50]; Zhu et al. [53]
HSE organization, responsibilities, and authorities	McDonald et al. [49]; Chang and Liang [7]; Qiu et al. [33]; Yan et al. [6]
Leadership and commitment	Zohar [42]; Niskanen [54]; Dedobbeleer and Béland [55]; Guldenmund [52]; Kang et al. [10]; Yan et al. [6]
Objectives	Niskanen [54]; Høivik et al. [50]; Yan et al. [6]
Incentives and accountability	Pashapour et al. [9]
Risk control	Isla Díaz and Díaz Cabrera [40]; Guldenmund [51]; Flin et al. [41]; Guldenmund [52]; Qiu et al. [33]; Yan et al. [6]; Zhu et al. [53]
Staff management and training education	Zohar [42]; Guldenmund [51]; Flin et al. [41]; Guldenmund [52]; Chang and Liang [7]; Kang et al. [10]; Zhu et al. [53]
Employee participation	Dedobbeleer and Béland [55]; Høivik et al. [50]
Implementation and supervision of medical health plans	Teo and Ling [56]; Chang and Liang [7]; Ilbahar et al. [11]
Self-inspection and rectification	Guldenmund [51]; Thomas Ng et al. [57]
Communication	Geller [43]; Niskanen [54]; Hale [44]; Guldenmund [51]; Guldenmund [52]; Chang and Liang [7]
Integration	Zohar [42]; Geller [43]; Niskanen [54]
Internal audit and management review	Chang and Liang [7]; Kang et al. [10]; Yan et al. [6]
Accident report, investigation, and management	McDonald et al. [49]; Thomas Ng et al. [57]; Chang and Liang [7]; Qiu et al. [33]; Yan et al. [6]; Zhu et al. [53]
Keep improving	Geller [43]; Niskanen [54]; Guldenmund [52]

**Table 2:** Brief description of six experts in the questionnaire presurvey.

Experts	Brief description
Expert 1	HSE Director and Head of Safety and Environmental Protection Department, holding a master's degree, with about 20 years of working experience in the safety engineering management field

Expert 2	Deputy Director of Safety and Environmental Protection Department, holding a master's degree in safety engineering, with about 20 years of working experience in freeway construction

Expert 3	Working in a safety production technology center, holding a master's degree, with over 10 years of working experience in the safety protection field

Expert 4	Working in China Classification Society, holding a bachelors' degree in structural engineering, with over 15 years of working experience in the safety evaluation of marine operations

Expert 5	A bridge segment safety director, holding a master's degree, with about 15 years of working experience in road and bridge construction and operation field

Expert 6	An island section safety director, holding a bachelors' degree in civil engineering, with over 20 years of working experience in the safety engineering management field

**Table 3:** Clusters of questions in the survey.

Indicators	Questions
Management system and procedures	The HSE system and procedures are in line with the actual project and are highly operable
	Institutions and procedures involve ergonomics and psychosocial environments
	Few redundant institutional procedures and complicated contents, and employees can comply with the system to carry out daily work

HSE organization, responsibilities, and authorities	HSE's full-time management agency plays an important role in HSE management
	Employees can clarify their HSE responsibilities
	Managers have ability to respond to HSE risks in special situations and take action timely

Leadership and commitment	Leaders participate in HSE management
	Leaders support HSE management
	Leaders attach importance to HSE management and psychological environment and balance the relationship between HSE management and construction period

Objectives	Management objectives are effective, measurable, and achievable
	Regularly assess the completion of the target and adjust the target according to the actual situation
	Incorporate the psychology into management objectives

Incentives and accountability	Set up a reasonable reward and punishment system with a combination of various rewards and punishment methods
	The reward and punishment system encourages employees to work with enthusiasm and reduce repetitive mistakes
	Reward individuals appropriately who have outstanding performance

Risk control	Conduct risk assessments regularly and monitor major sources of risk dynamically
	Participate in risk management comprehensively
	Consider the risk of social psychology and ergonomics in risk source identification

Staff management and training education	Formulate a reasonable training plan for different positions and combine it with the assessment system to ensure the training effect
	Strictly manage employee accessibility and reduce staff mobility
	Training and education involve ergonomics and psychosocial environments

Employee participation	Participate in HSE management activities such as risk source identification and accident investigation
	Think that work is indispensable for the success of HSE management
	Be able to consciously discover the HSE risks that exist around you and promptly give feedback to relevant personnel

Implementation and supervision of medical health plans	Formulate a health plan for different positions
	Deploy special personnel to supervise or set dynamic monitoring for dangerous positions
	Wear personal protective equipment and check regularly

Self-inspection and rectification	Conduct regular inspection activities
	Correct the unqualified behavior found in the inspection and track the rectification results
	Repetitive and habitual violations have been reduced

Communication	Communicate with grassroots employees regularly, understand their physical and psychological status, and listen carefully to their suggestions or opinions
	Communicate with leaders timely and make recommendations to leaders based on your professional knowledge
	Communicate with colleagues timely, share lessons learned from HSE management, and improve HSE management

Integration	Integrate safety management with occupational health and environmental protection
	Integrate ergonomics and the social psychology with HSE management
	Integrate HSE management with other engineering management efforts

Internal audit and management review	Conduct an internal audit and management review work regularly
	Take timely action on identified issues and track improvement results
	In the internal audit and management review, consider the construction of the social-psychological environment and the application of the ergonomics in the HSE management system

Accident report, investigation, and management	Consideration of the impact of ergonomics and social psychology on accidents in accident investigation and cause analysis
	Ergonomics and psychosocial factors are taken into account in corrective and preventive measures after an accident
	Compared with similar projects, the accident rate is significantly reduced

Keep improving	Continuous improvement systems are compliant and effective
	Introduce advanced management methods to improve HSE management performance
	Improve HSE management procedures based on lessons learned in practice

Job satisfaction	Be satisfied with the physical environment such as equipment and facilities, air quality, and lighting conditions in the workplace
	Workload and HSE responsibilities are allocated reasonably
	Be satisfied with your position and work content

HSE awareness	The social and psychological environment in the organization is good, and the attitude of employees is positive
	HSE management is very important for organizational management
	The HSE management of this project has achieved good effects

Work stress	Have no adverse emotions such as anxiety due to stress in the work
	Daily work can be completed within the specified time, and there is very little overtime
	Have no abnormal headaches, dizziness, and other symptoms after daily work

**Table 4:** The detailed demographics of respondents.

Demographic factor	Range	Number	Percentage
Age (year)	<18	0	0
	18–25	5	5.2
	26–35	18	18.8
	36–45	39	40.6
	46–55	26	27.1
	>55	8	8.3

Gender	Male	82	85.4
	Female	14	14.6

Experience (year)	<5	7	7.3
	5–10	13	13.5
	11–15	31	32.3
	16–20	27	28.1
	>20	18	18.8

Department	HSE committee	32	33.3
	Safety and Environmental Protection Department	22	22.9
	Operation Management Department	12	12.5
	Transportation Engineering Department	25	26.0
	General Affairs Department	5	5.2

Position	HSE administrators	42	43.8
	Supervisors	8	8.3
	Security officers (including machinery, shipping, and site)	23	24.0
	Engineers	12	12.5
	Consultants	3	3.1
	Others	8	8.3

HSE: health, safety, and environment.

**Table 5:** 102 kinds of neural network models and their MAPE.

Number	Training function	Transfer function		Job satisfaction		Work stress		HSE awareness	
				Numbers	MAPE	Numbers	MAPE	Numbers	MAPE
1	Trainlm	Logsig	Purelin	14	0.0559	20	0.0752	14	0.0965
2	Trainb	Logsig	Purelin	14	0.0597	44	0.0814	32	0.0918
3	Trainbfg	Logsig	Purelin	10	0.0516	45	0.0669	12	0.0836
4	Trainbr (sse)	Logsig	Purelin	2	0.0741	2	0.1286	32	0.1074
5	Trainc	Logsig	Purelin	37	0.0558	32	0.0705	25	0.0754
6	Traincgb	Logsig	Purelin	49	0.0519	27	0.0779	6	0.0999
7	Traincgf	Logsig	Purelin	23	0.0626	21	0.0687	11	0.0802
8	Traincgp	Logsig	Purelin	8	0.0507	42	0.0692	16	0.0836
9	Traingd	Logsig	Purelin	16	0.0514	24	0.0658	22	0.0741
10	Traingda	Logsig	Purelin	35	0.0613	32	0.0610	20	0.0830
11	Traingdm	Logsig	Purelin	6	0.0510	32	0.0615	12	0.0971
12	Traingdx	Logsig	Purelin	20	0.0476	18	0.0856	32	0.0832
13	Trainoss	Logsig	Purelin	6	0.0542	29	0.0721	37	0.0847
14	Trains	Logsig	Purelin	13	0.0508	14	0.0677	39	0.0908
15	Trainscg	Logsig	Purelin	14	0.0539	48	0.0650	41	0.0730
16	Trainr	Logsig	Purelin	12	0.0580	26	0.0646	38	0.0911
17	Trainrp	Logsig	Purelin	34	0.0530	24	0.0674	42	0.0858
18	Trainlm	Tansig	Purelin	8	0.0724	8	0.0894	5	0.1332
19	Trainb	Tansig	Purelin	6	0.0516	5	0.0970	12	0.1114
20	Trainbfg	Tansig	Purelin	19	0.0529	45	0.0614	**23**	**0.0713**
21	Trainbr (sse)	Tansig	Purelin	34	0.0734	18	0.1061	22	0.1099
22	Trainc	Tansig	Purelin	2	0.0634	2	0.0907	6	0.0987
23	Traincgb	Tansig	Purelin	14	0.0499	1	0.0679	13	0.1028
24	Traincgf	Tansig	Purelin	3	0.0688	14	0.0659	4	0.1028
25	Traincgp	Tansig	Purelin	4	0.0695	22	0.0875	13	0.0953
26	Traingd	Tansig	Purelin	3	0.0661	31	0.0854	5	0.1075
27	Traingda	Tansig	Purelin	31	0.0512	4	0.0934	6	0.0879
28	Traingdm	Tansig	Purelin	5	0.0522	**13**	**0.0572**	3	0.0964
29	Traingdx	Tansig	Purelin	**14**	**0.0439**	17	0.0801	7	0.0817
30	Trainoss	Tansig	Purelin	49	0.0521	7	0.0871	3	0.0988
31	Trains	Tansig	Purelin	3	0.0654	32	0.0760	12	0.1057
32	Trainscg	Tansig	Purelin	9	0.0637	47	0.0749	8	0.1006
33	Trainr	Tansig	Purelin	33	0.0645	15	0.0847	8	0.0952
34	Trainrp	Tansig	Purelin	16	0.0631	18	0.0630	2	0.1067

*Notes*. Bold values mean selected neural networks with minimal MAPE

**Table 6:** Indicators' description statistic.

Indicators	Mean	Minimum value	Maximum value	Cronbach's alpha	CVR
HSE organization, responsibilities, and authority	0.98	0.3	1	0.833	0.60
Employee participation	0.97	0.53	1	0.855	0.60
Risk control	0.97	0.42	1	0.806	0.47
Management system and procedures	0.97	0.15	1	0.803	0.66
Implementation and supervision of medical health plans	0.97	0.57	1	0.879	0.54
Integration	0.96	0.7	1	0.906	0.43
Leadership and commitment	0.96	0.6	1	0.848	0.60
Staff management and training education	0.94	0.66	1	0.802	0.80
Communication	0.94	0.6	1	0.726	0.73
Objectives	0.94	0.62	1	0.808	0.49
Self-inspection and rectification	0.94	0.31	1	0.784	0.62
Incentives and accountability	0.92	0.19	1	0.792	0.48
Internal audit and management review	0.91	0	1	0.847	0.44
Keep improving	0.91	0.49	1	0.831	0.49
Accident report, investigation, and management	0.8	0.31	1	0.750	0.51
Work stress	0.81	0.6	1	0.906	0.73
HSE awareness	0.67	0.4	0.9	0.883	0.62
Job satisfaction	0.55	0.3	0.8	0.879	0.80

**Table 7:** Correlation of the effectiveness of various factors.

Correlation coefficient	Job satisfaction effectiveness	Work stress effectiveness	HSE awareness effectiveness
Job satisfaction effectiveness	1		
Work stress effectiveness	0.119	1	
HSE awareness effectiveness	−0.052	−0.066	1

**Table 8:** Paired sample *t*-test results.

Deleted variable	Mean	95% confidence interval		Two-tailed paired *t*-test *p* value (sig.)	Test results	Impact on effectiveness
		Lower limit	Upper limit			
Management system and procedures	−0.06685	−0.07304	−0.06066	0	*μ*_*F*_ = *μ*_*i*_	No significant impact
HSE organization, responsibilities, and authority	−0.00794	−0.01498	−0.00090	0.027	*μ*_*F*_ = *μ*_*i*_	No significant impact
Leadership and Commitment	0.08257	0.07340	0.09175	0	*μ*_*F*_ = *μ*_*i*_	No significant impact
Objectives	−0.16297	−0.17197	−0.15398	0	*μ*_*F*_ = *μ*_*i*_	No significant impact
Incentives and accountability	−0.15791	−0.16670	−0.14911	0	*μ*_*F*_ = *μ*_*i*_	No significant impact
Risk Control	0.00272	−0.00334	0.00879	0.375	*μ*_*F*_ > *μ*_*i*_	Positive impact
Staff management and training education	−0.16425	−0.17395	−0.15456	0	*μ*_*F*_ = *μ*_*i*_	No significant impact
Employee participation	0.00245	−0.00498	0.00987	0.514	*μ*_*F*_ > *μ*_*i*_	Positive impact
Implementation and supervision of medical health plans	0.03124	0.02349	0.03898	0	*μ*_*F*_ = *μ*_*i*_	No significant impact
Self-inspection and rectification	−0.01976	−0.03035	−0.11867	0	*μ*_*F*_ = *μ*_*i*_	No significant impact
Communication	−0.13136	−0.14405	0.11867	0	*μ*_*F*_ = *μ*_*i*_	No significant impact
Integration	0.18880	0.18060	0.19699	0	*μ*_*F*_ = *μ*_*i*_	No significant impact
Internal audit and management review	−0.02351	−0.03207	−0.01496	0	*μ*_*F*_ = *μ*_*i*_	No significant impact
Accident report, investigation, and management	0.20152	0.19308	0.20997	0	*μ*_*F*_ = *μ*_*i*_	No significant impact
Keep improving	0.15542	0.14796	0.16288	0	*μ*_*F*_ = *μ*_*i*_	No significant impact
Job satisfaction	−0.00979	−0.01987	0.00029	0.057	*μ*_*F*_ < *μ*_*i*_	Negative impact
Work stress	0.04533	0.03421	0.05645	0	*μ*_*F*_ = *μ*_*i*_	No significant impact
HSE awareness	0.06851	0.25249	0.28026	0	*μ*_*F*_ = *μ*_*i*_	No significant impact

## Data Availability

The data used to support the findings of this study are available from the corresponding author upon request.
